# Determination of biochemical and histopathological changes on testicular and epididymis tissues induced by exposure to insecticide Imidacloprid during postnatal development in rats

**DOI:** 10.1186/s40360-023-00709-3

**Published:** 2023-11-27

**Authors:** Amina Sardar, Mehwish David, Sarwat Jahan, Tayyaba Afsar, Aneela Ahmad, Asad Ullah, Ali Almajwal, Huma Shafique, Suhail Razak

**Affiliations:** 1 Reproductive Physiology Laboratory, Department of Zoology, Quaid-I-Azam University, Islamabad, Pakistan; 2 Department of Community Health Sciences, College of Applied Medical Sciences, King Saud University, Riyadh, Saudi Arabia; 3 Institute of Cellular Medicine, Newcastle University Medical School, Newcastle University, Upon Tyne, United Kingdom

**Keywords:** Imidacloprid, Neonates, Histopathological changes, Oxidative stress, Reproductive toxicity

## Abstract

**Background:**

Imidacloprid is a neonicotinoid insecticide belonging to the chloronicotinyl nitroguanidine chemical family. Toxicity of IMD for mammals in scientific studies has shown high mutagenic, immunotoxic, teratogenic and neurotoxic effects. The present study was designed to assess the toxic effects of imidacloprid (IMD) on the testicular and epididymis tissues as well as testosterone levels of neonatal male rats.

**Methods:**

Neonatal male rats from postnatal day (PND) 1 to PND 26 were consecutively administered with different concentrations of IMD (1, 5 and 10 mg/kg) subcutaneously. The effect of IMD on body and organ weight, lipid profile, histopathological alterations, oxidative stress and altered testosterone levels were assessed in the testis and plasma.

**Results:**

The results of body weight gain showed a significant difference in group 4 (10 mg/kg) animals as compared to the control. A significant increase in total cholesterol and triglycerides, while a decrease in high-density lipoprotein concentrations was evident. Similarly, a significant decrease in concentrations of antioxidant enzymes (CAT and SOD) among all the IMD-treated groups was evident, when compared to the control. Increased production of ROS was also noticed in the highest-dose treatment group. Further, we observed that IMD-treated rats indicated histopathological changes in the testis and epididymis along with a significant decrease in the plasma testosterone concentrations among IMI-treated groups in contrast to the control. Histological examination of the testis of IMD-treated neonatal male rats also showed decreased spermatogenesis in the treated groups when compared to the control. Furthermore, an increase in lumen diameter and a decrease in epithelial height of seminiferous tubules were also observed in IMD-treated rats in comparison with the control.

**Conclusion:**

It is concluded that sub-chronic exposure to IMD in neonatal male rats may induce histopathological changes in reproductive tissues and damage normal testicular functions via inducing oxidative stress, decrease in body weight, disturbing normal blood lipid profile and testosterone concentration. IMD exposure can induce pathophysiological effects calls for further evaluation of this widely used insecticide.

## Introduction

Over the few past decades, much evidence has shown that many toxicants in the environment have deleterious effects on male and female reproduction. Pesticide toxicity is a global concern for non-target species. Pesticides can be any substance or combination of different substances used to eradicate unwanted insects like disease vectors among human beings and animals, fungi, or weeds to increase food production and help the production process, transport, marketing or storage of the food materials [1]. Pesticides at the time may also destroy agricultural products and soil [2]. Humans these days are exposed to different pesticides by air, water and food [3]. Pesticide exposure at times also increases the interest of the general population in potential and promising hazards to human health including chronic and acute poisonings [4]. Many pesticides possibly act like endocrine-disrupting chemicals (EDCs) and induce toxicity because of having a similar structure with the different endogenous hormones of the body [5, 6]. Hence, some of the most commonly used pesticides can bind to the receptors of different hormones and affect normal activity by leading to several disorders [7]. Pesticide exposure has also been observed to interrupt the process of spermatogenesis by damaging the different compartments of the testis and sperm morphology [8]. Studies in the past have also shown that exposure to pesticides can affect the structure and morphology of seminiferous tubules resulting in altered spermatogenesis [9–12].

Insecticides are heterogeneous groups of chemicals having the potential of killing pests by piercing into their bodies through oral, dermal, and respiratory routes [13]. Neonicotinoids are among the most currently used insecticides worldwide (Jeschke et al., 2011). Seven types of neonicotinoid pesticides are currently available in the market namely clothianidin, acetamiprid, nithiazine, imidacloprid, nitenpyram, thiamethoxam, and thiacloprid [14]. Neonicotinoid insecticides became successful in 1991 when Bayer Crop Science (BCS) launched forerunner insecticide imidacloprid (IMD) which has been the largest-selling insecticide in the world (Jeschke et al., 2010). Recent studies have shown that globally, the usage rate of neonicotinoids is about 20–24% including IMD, whose rate of use is growing 10%/year only in Bangladesh [15, 16]. IMD belongs to the neonicotinoid class of pesticides and acts as a nicotine acetylcholine receptors agonist (nAChRs) in insects [17]. Toxicity of IMD for mammals in scientific studies has shown high mutagenic [18], immunotoxic, teratogenic and neurotoxic effects [19, 20]. IMD is also taken up into growing plant tissues where it has broad and long-lasting toxicity on a variety of plants as well as insect pests which feed on it [21, 22]. In addition, studies indicated that IMD and acetamiprid exposure during *in-vitro* conditions adversely change the early embryonic development and sperm functions, morphological alterations and DNA damage in rodents [23, 24]. Moreover, IMD exposure during the gestational period led to fetal deformities of young male rats by altering their neurobehavioral [25].

Although there are several toxic effects of IMD regarding the retention in fruits, vegetables, crops and physical contact in pets, its high usage globally is alarming for both human and wildlife exposure [26]. However, very little literature is available on the neonatal exposure of male rats to different doses of IMI to determine the reprotoxic potential of this pesticide in neonates. Therefore, the current study was designed to investigate the possible detrimental effect of IMD on the biochemical profile and functioning of the reproductive system of neonatal male rats exposed consecutively from PND 1 to PND 26.

## Materials and methods

### Animals, chemicals and experimental design

The present study was conducted in the Reproductive Physiology Laboratory, Department of Zoology, Quaid-i-Azam University, Islamabad, Pakistan. Neonatal Sprague Dawley male rats (4–5 g of weight) were taken from animal house, Quaid-i-Azam University, Islamabad. Before the start of the experiment, four wooden breeding cages were separated, and four adult female rats were placed in each breeding cage with two adult male rats. Animals were fed with a pelleted diet and access to water *ad labitium.* After ten days, adult males were separated from females. Pregnant females were reared singly till the birth of pups on gestational day 22 (GD-22). The day of birth of the pups was deliberated as the post-natal day (PND) 1. The total number of pups included in the study was 40 (*N* = 10 pups/group). On PND 27, animals were given anaesthesia, provided via intraperitoneal injection of a ketamine/xylazine mixture (75/2.5 mg/kg, respectively). The study is reported under ARRIVE guidelines [27]. All the experimental protocols and animal handling were evaluated and approved (BAS0256) by the Department of Zoology by the ethical committee of the Department of Zoology, Quaid-i-Azam University Islamabad, Pakistan. All methods were performed following the relevant guidelines and regulations.

The chemical used was technical grade IMD and was purchased from Sigma Aldrich, Germany. IMD doses were selected based on their LD 50, which is reported to be 450 mg/kg body weight (Bomann, 1989). Three doses of IMD 1 mg/kg, 5 mg/kg and 10 mg/kg were selected after a detailed literature review [28, 29] to study the dose-dependent toxic effects on testicular tissue histology and biochemical parameters from PND1 to PND 26. The male pups were assigned into four different groups. For the stock solution, 25 mg of IMD was dissolved in 50 ml of corn oil. Three different solutions of IMD with concentrations of 1mg/kg, 5mg/kg, and 10mg/kg were prepared from this stock solution. Group one was assigned as control and was given corn oil via a subcutaneous (S.C) route from PND 1 to PND 26. The second group (G1) was served with 1 mg/kg of IMD, while, groups three (G2) and four (G3) were treated with 5 mg/kg and 10 mg/kg of IMD in corn oil from PND 1 to PND 26. On PND 27, animals were anesthetized using combined intraperitoneal injection of ketamine (75 mg/kg) and xylazine (2.5 mg/kg) [30]. Anesthetized rats were secured in a supine position and thoracotomy is performed to assure euthanasia. Euthanasia method was performed in accordance with AVMA guidelines [31]. Blood sample was withdrawn via cardiac puncture and dispensed in heparinized tubes, and then centrifuged at 3000rpm for 15 min and plasma was separated and kept at -20 °C till hormonal analysis. Reproductive organs (testis, epididymis, prostate gland and seminal vesicle) were cleaned of fats, washed, blotted on filter paper and weighed. From each rat, one testis was stored in the freezer (-80 °C) for estimation of biochemical and oxidative stress-related parameters. The other testes and epididymis were allowed to fix in 10% formalin until processed for histological examination.

### Body and organ weights

Body weights of all the male pupe were noted using Sarotoreious Digital Balance on a postnatal day (PND) 1, 8, 16, 22 and at PND 26, however, here only weights taken at the start and end of the experimentation are mentioned to estimate the final weight gain.

### Biochemical analysis

#### Lipid profile

Plasma levels of triglyceride, total cholesterol, and low-density lipoprotein (LDL) were checked using commercially available AMP diagnostic kits (AMEDA laboratory diagnostic Gmbh, Austria) by following the manual provided by the manufacturer using Picco five chemistry analyzer (AMP diagnostic).

#### HDL Cholesterol level

HDL levels of cholesterol were estimated using the formula:

$$\mathrm{HDL}\;\mathrm{cholesterol}\;=\;\mathrm{Total}\;\mathrm{cholesterol}-\mathrm{HDL\ cholesterol}-(\mathrm{Triglycerides}/5)$$

### Antioxidant enzymes quantification

Tissues collected from the study were further processed for the oxidative stress markers and antioxidant enzymes. Tissues were homogenized with an automatic homogenizer in phosphate buffer saline (PBS) and centrifuged at 30,000 rpm for 30 min. After the centrifugation, the supernatant was removed and used for the hormonal analysis, protein estimation and antioxidant enzymes.

#### Catalase (CAT)assay

The catalase activity was determined by the method used by afsar and colleagues [32], and the change in the absorbance due to H_2_O_2_ was measured in the tissues. In this assay, 50 µl homogenate was diluted in 2 ml of phosphate buffer with a pH of 7.0. After thorough mixing, the absorbance was read at 240 nm with an interval of 15 s and 30 s. Change in the absorbance of 0.01 as unit/min was defined as one unit of CAT.

#### Superoxidase dismutase (SOD) assay

Superoxide dismutase (SOD) activity was estimated by a previously established method by Afsar et al. (2020) and Kakkar et al. (1984) [33, 34]. In this assay, the amount of chromogen formed was measured at 560 nm. The results were expressed in units/mg of protein.

#### Lipid peroxidation assay

Oxidative stress induced by reactive oxygen species (ROS) was estimated by the method of Hayashi and coworkers [35], and for the presentation of mean values, the assay was repeated multiple times. In this assay, 5 ml of H_2_O_2_ standards and the homogenate were mixed with 140 ml of sodium acetate buffer with pH 4.8 in 96 wells plates and incubated at 37 °C for 5 min. Following incubation, 100 ml of N, N′-diethyl-1,4-phenylenediamine (DEPPD) and ferrous sulfate mixed sample was added in each well with a ratio of 1:25 and were incubated at 37 °C for 1 min. With an interval of 15 s for 3 min, the absorbance was read at 505 nm at the microplate reader.

### Hormone analysis

Testosterone concentration was quantified using commercially available enzyme immunoassay (EIA) kits (Biocheck Inc, USA, catalogue number: 500396). The assay procedure was done following the guideline provided by the manufacturers using a microplate reader at a wavelength of 450 nm. The minimum sensitivity of the assay was 0.05 ng/ml as provided in the kit.

### Tissue histology

Testicular tissues (Testes and Epididymis) were fixed in formalin for 48 h. Dehydrated with different grades of alcohol and cleared with the help of xylene. The paraffin Sects. (5 µm) were cut and stained with hematoxylin and eosin to assess standard histology and morphometry. Testicular sections from 10 to 20 per group were digitized under Leica Microscope (New York Microscope Company) equipped with a digital camera (Canon, Japan).

For the morphometry, the seminiferous tubule diameter and seminiferous tubule epithelial height of testicular tissue were measured. Shortly, a picture of the known distance in micrometres was used for setting the scale and conversion of values from pixels to micrometres. The area of different sections was calculated in μm^2^ using Image J software following the method described previously [30, 36]. From 20X images, 30 pictures per animal were selected and the area of the seminiferous tubule and interstitial space was calculated by planimetry using Image J software (Image J2 × 2.1.4.7 Image 1.0 Wayne Rasband National Institutes of Health, USA). Several different cell types (spermatids, spermatogonia and spermatocytes) and the area of seminiferous tubules and interstitial space were determined by the free selection tool of the software. A comparison of different groups with control was done using the image J2X software package program. The area percentage (%) was attained by the formula:

% Area of seminiferous tubule (AS) = [As x 100/T], where T is the total area of the field. The percentage of the mean area was analyzed for a comparison between the treated and control groups.

### Statistical analysis

The animal number for the current study was calculated by the resource equation method [37] by using the following formula:

$$\mathrm{E }=\text{Total number of animals }-\text{Total number of groups}$$

Here, E is the degree of freedom of analysis of variance (ANOVA). The value of E should lie between 10 and 20 to increase the chance of getting a more significant result. As this method is based on ANOVA, it applies to all animal experiments [38].

In the present study, we made four groups with ten animals each. So,

$$\mathrm{E }=\left(10 \times 4\right)-4$$

E = 40–4 = 36, This sample size is adequate as the chances of death of animals cannot be ignored.

The normality of distribution was tested with the Kolmogorov–Smirnov test. The data obtained for organ weight and oxidative stress parameters were normally distributed. Therefore, statistical significance between different treatment groups for various parameters was analyzed with One-way ANOVA, followed by *post-hoc* Tukey’s HSD test. All the values were represented as Mean ± SEM. One-way ANOVA of variance was performed using Graph Pad Prism 9 software The probability value (*P* < 0.05) was measured as statistically significant.

## Results

### Body and organ weights

Initial body weight, final body weight and body weight gain in all the treated groups with IMD and control are presented in Table 1. There was observed no noteworthy body weight gain after 26 days of exposure among the G1 (1 mg/kg) and G2 (5 mg/kg) groups as compared to the control. However, there was a notable weight reduction was observed in the G3 (10 mg/kg) group when compared with the control (Table 1). A noticeable decrease was observed in the testicular weight of all the IMD-treated groups as compared to the control. Similarly, a little reduction in the weight of accessory organs including the epididymis, seminal vesicle and prostate was noticed in all IMD-exposed groups, however, the decline is not statistically significant compared with the control group.

**Table 1:** Effects of Imidacloprid on body and organ weights after prenatal exposure from PND1-PND26 (Mean ± SEM)

Treatments	PND1	PND27	Body weight gain	Testis Paired weight (g)	Prostate gland (g)	Seminal vesicle (g)	Epididymis (g)
C (0 mg/kg)	4.40 ± 0.25	33.60 ± 1.13	30.60 ± 1.49	0.74 ± 0.19	0.04 ± 0.01	0.34 ± 0.19	0.35 ± 0.02
G1 (1 mg/kg)	4.60 ± 0.25	30.60 ± 1.08	27.25 ± 0.97	0.57 ± 0.01	0.06 ± 0.01	0.03 ± 0.01	0.32 ± 0.01
G2 (5 mg/kg)	4.40 ± 0.25	27.80 ± 1.43	26.50 ± 0.34	0.39* ± 0.17	0.05 ± 0.01	0.01 ± 0.02	0.31 ± 0.01
G3 (10 mg/kg)	4.60 ± 0.25	25.80 ± 0.67	24.00 ± 1.65^a**^	0.25^ab***^ ± 0.01	0.02 ± 0.01	0.09 ± 0.07	0.28 ± 0.03

^*^, **, *** indicate significant differences at probability values *P* < 0.05, *P* < 0.01 and *P* < 0.001 compared to the control (ANOVA followed by Tukey’s comparison test)

a = vs control

b = vs G1

### Effect on antioxidant and oxidative stress biomarker enzymes

**Fig. 1:**
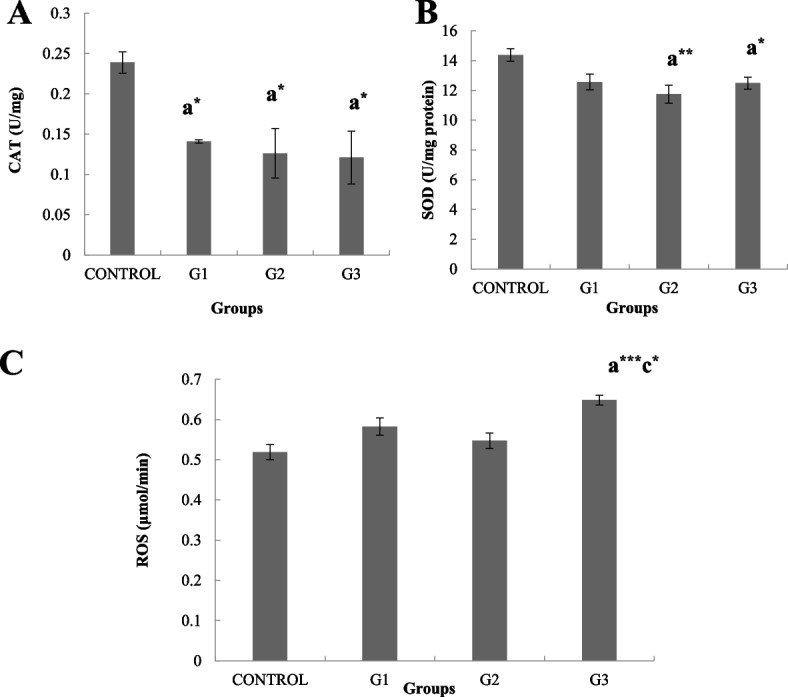
The figure shows the antioxidant /oxidative stress parameters in testicular tissues of neonatal rats that were treated with IMI from PND1 to PND 26. A dose-dependent decrease in (**A**) Catalase (U/mg) and (**B**) Sodium dismutase (U/mg protein), and an increase in (**C**) reactive oxygen species number (µmol/min) were noted. *, **, *** indicate significant differences at probability values P < 0.05, *P* < 0.01 and *P* < 0.001 compared to the control (ANOVA followed by Tukey’s comparison test). a = vs control, b = vs G1, c = vs G2

A significant decrease (*p* < 0.05) in CAT activity in all IMD groups was observed when compared to the control (Fig. 1). However, there was no significant difference in SOD level was observed in the G1 group (1 mg/kg) when compared with the control. Whereas, there was seen a noticeable decrease in SOD activity in the G2 (5 mg/kg) (*p* < 0.01) and G3 (10 mg/kg) (*p* < 0.05) groups when comparison was done with the control. G1 (1 mg/kg).

The results of oxidant analysis showed a significant increase (*p* < 0.01) in the production of reactive oxygen species number among the G3 (10 mg/kg) group when compared to the control. However, there was no significant difference observed when G1 (1 mg/kg) and G1 (5 mg/kg) groups were compared with the control (0 mg/kg).

### Hormone analysis

The hormonal analysis presented a significant reduction (*p* < 0.05) in plasma testosterone concentrations among G2 and G3 groups as a result of IMD treatment when compared with the control. In G1 (1 mg/kg) no significant changes were observed when treated groups are compared to each other (Table 2).

**Table 2:** Mean ± SEM of plasma testosterone concentration various treatment groups

Groups	Plasma Testosterone Concentration (ng/dl)
C	0.42 ± 0.04
G1	0.32 ± 0.02
G2	0.30 ± 0.03^*^
G3	0.25 ± 0.02^**^

Values are represented as mean ± SEM

^*^, **, *** indicate significant differences at probability values *P* < 0.05, *P* < 0.01 and *P* < 0.001 compared to the control (ANOVA followed by Tukey's comparison test)

### Lipid profile

**Fig. 2:**
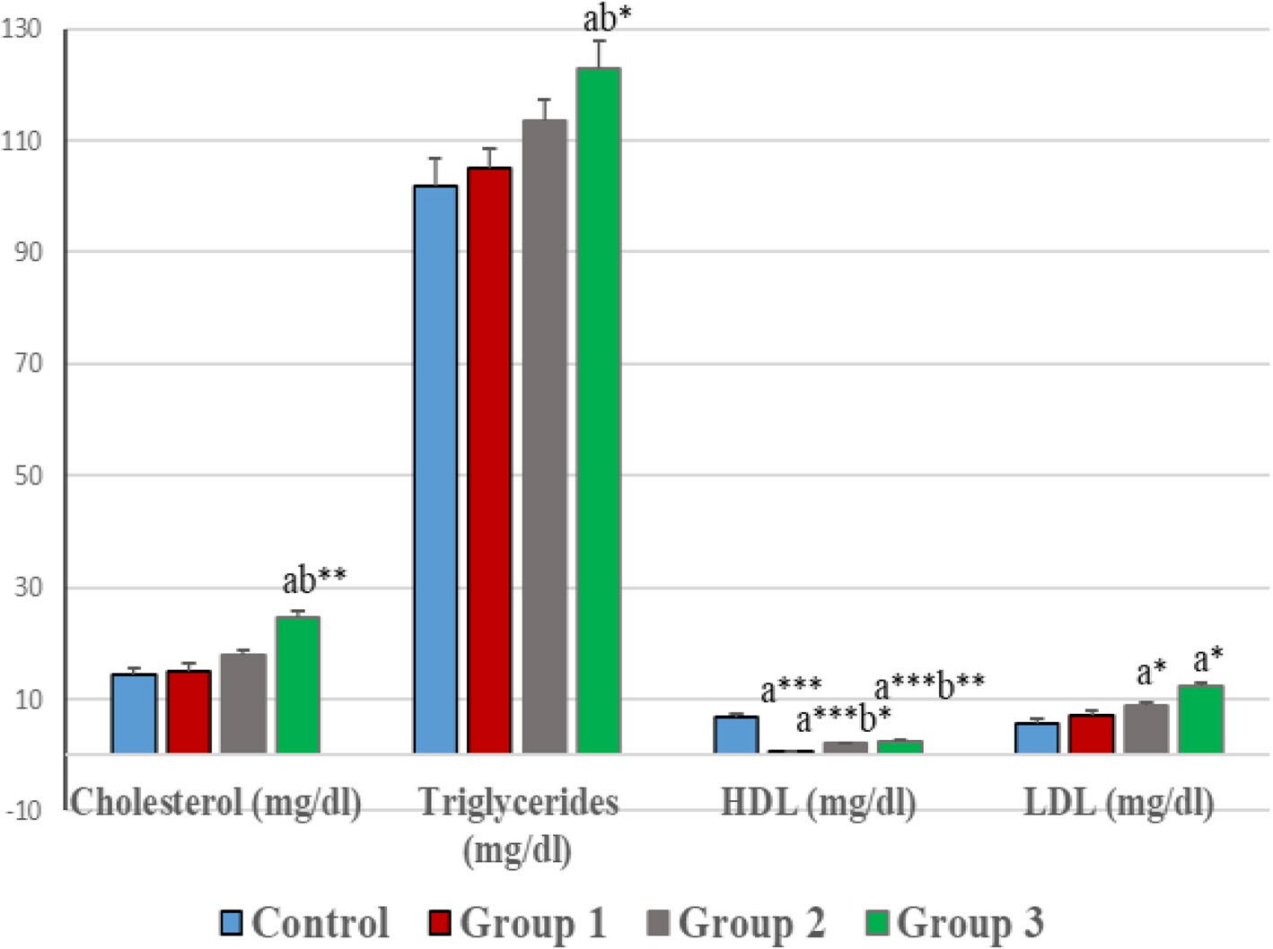
Effect of various treatments on Lipid Profile. Values are represented as mean ± SEM. *, **, *** indicate significant differences at probability values *P* < 0.05, *P* < 0.01 and *P* < 0.001 compared to the control (ANOVA followed by Tukey's comparison test). a = vs control, b = vs G1

No significant difference was observed in the concentration of total cholesterol among the G1 (1 mg/kg) and G2 (5 mg/kg) groups when compared with the control group (Fig. 2). A significant increase (*p* < 0.01) in total cholesterol level was seen in G3 (10 mg/kg) as compared to the control. Similarly, a significant (*p* < 0.01) high level of cholesterol was seen in G3 (10 mg/kg), when compared with G1 (1 mg/kg) and G1 (5 mg/kg) groups.

Similarly, significantly higher levels of triglycerides (*p* < 0.05) were measured in the G3 (10 mg/kg) group while G1 (1 mg/kg) and G2 (5 mg/kg) groups showed no significant change when compared with the control. No remarkable difference within groups was evident.

Significant (*p* < 0.001) low levels of HDL were observed in all the IMD-treated groups (G1, G2, G3) as compared to the control. Similarly, significant (*p* < 0.05, *p* < 0.01) reduction was obtained in G2 (5 mg/kg) and G3 (10 mg/kg) in contrast to G1 (1 mg/kg) group.

A significantly (*p* < 0.05) raised level of LDL was noticed in G2 (5 mg/kg) and G3 (10 mg/kg) as compared to the control. In contrast, no significant difference in LDL levels was experienced within groups.

### Histopathology

#### Testis

**Fig. 3:**
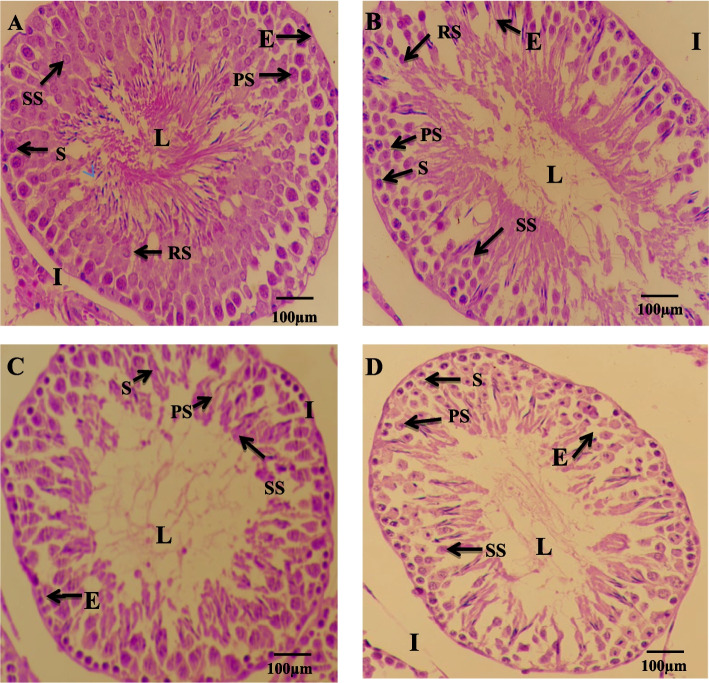
Photomicrographs of HE-stained testis from PND 26 juvenile male rats. Dense tubules, lumen congested with normal germ cells along normal epithelium shown in (**A**) Control; (**B**) IMD (1 mg/kg) showing tubules with minimal degenerated epithelial layer and increased tubular diameter and larger lumen space (**C**) IMD (5 mg/kg) illustrating wider lumen, compact tubules with increased epithelial height, reduced number of spermatocytes and arrested spermatogenesis (**D**) IMD (10 mg/kg); showing damage of epithelium, lumen wider with arrested spermatogenesis. Spermatogonia (S), Primary spermatocytes (PS), Secondary spermatocytes (SS), Round spermatocytes (RS), Lumen (L), Basal compartment (**B**), Interstitial space (I), Epithelium (**E**). Pictures were taken at Magnification 40X

Testis and epididymis morphological changes in the area of seminiferous tubule and tubular lumen diameter and epithelial height after 26 days of exposure are presented in Table 3. Histological examination of testicular tissue demonstrated a normal morphology with closely spaced seminiferous tubules enclosed by a thick layer of tunica albuginea that show normal spermatogenesis as shown in Fig. 3 (A). Thick stratified germinal epithelium presenting normal stages of spermatogenesis was evident with a lumen filled with mature spermatozoa. Histological analysis of the testis of IMD-treated neonatal male rats a reduced number of spermatocytes with arrested spermatogenesis in all the treated groups when compared to the control. A highly significant decrease (*p* < 0.001) in tubular diameter of seminiferous tubules were observed in all IMD treated group (1 mg/kg, 5 mg/kg, 10 mg/kg) when compared with control due to focal impairment of seminiferous tubules (Fig. 3B, C and D). Similarly, a highly significant decrease (*p* < 0.001) in tubular diameter was observed in G2 and G3 as compared with G1. However, no significant difference was seen in tubular diameter when G2 is compared with G3. A highly significant (*p* < 0.001) increase was noticed in the lumen diameter of G1 and G2 as compared with the control. However, when G1 and G3 were compared, a significant difference (*p* < 0.001) was noticed in tubular lumen diameter. A similar significant difference (*p* < 0.001) was detected when G2 is compared with G3 (Table 3). A significant decrease (*p* < 0.001) was detected in epithelial height in G2 and G3 groups as compared to the control as well as G1. A significant (*p* < 0.05) decrease was also seen in G3 in comparison to G2.

**Table 3:** Effect of Imidacloprid on histomorphometry in neonatal male rats

Treatments	Seminiferous tubule diameter(µm)	Tubular lumen diameter(µm)	Epithelial height(µm)
C (0 mg/kg)	99.71 ± 2.67	38.04 ± 1.10	21.54 ± 0.32
G1 (1 mg/kg)	89.23 ± 2.14 ^a**^	47.06 ± 1.99^a**^	20.62 ± 0.25
G2 (5 mg/kg)	77.38 ± 1.81^ab***^	49.09 ± 1.98^a***^	18.92 ± 0.30^ab***^
G3 (10 mg/kg)	74.82 ± 1.34 ^ab***^	72.27 ± 2.61^abc***^	17.70 ± 0.30^ab***c*^
Caput Epididymis			
Groups	**Duct diameter (µm)**	**Lumen diameter (µm)**	**Epithelial height (µm)**
C (0 mg/kg)	64.17 ± 2.72	56.90 ± 1.55	17.65 ± 0.21
G1 (1 mg/kg)	54.12 ± 2.97	44.69 ± 1.45^a***^	14.94 ± 0.34^a***^
G2 (5 mg/kg)	57.43 ± 3.10	47.97 ± 1.35^a***^	13.62 ± 0.27^a***b**^
G3 (10 mg/kg)	59.19 ± 2.82	37.90 ± 1.16^ac***b**^	13.32 ± 0.30^ab***^
Cauda Epididymis			
Groups	**Duct diameter (µm)**	**Lumen diameter (µm)**	**Epithelial height (µm)**
C (0 mg/kg)	67.45 ± 3.19	30.68 ± 0.64	14.96 ± 0.44
G1 (1 mg/kg)	58.23 ± 3.42	28.12 ± 0.90	13.14 ± 0.43^a***^
G2 (5 mg/kg)	61.49 ± 3.14	27.16 ± 1.01^a*^	13.33 ± 0.41^a***^
G3 (10 mg/kg)	48.39 ± 1.51^a***c**^	22.59 ± 0.75^ab***c**^	12.97 ± 0.21^a***^

^*^, **, *** indicate significant differences at probability values *P* < 0.05, *P* < 0.01 and *P* < 0.001 compared to the control (ANOVA followed by Tukey's comparison test)

^a=^ vs control

^b^^=^ vs G1, ^c=^ vs G2

### Caput epididymis

**Fig. 4:**
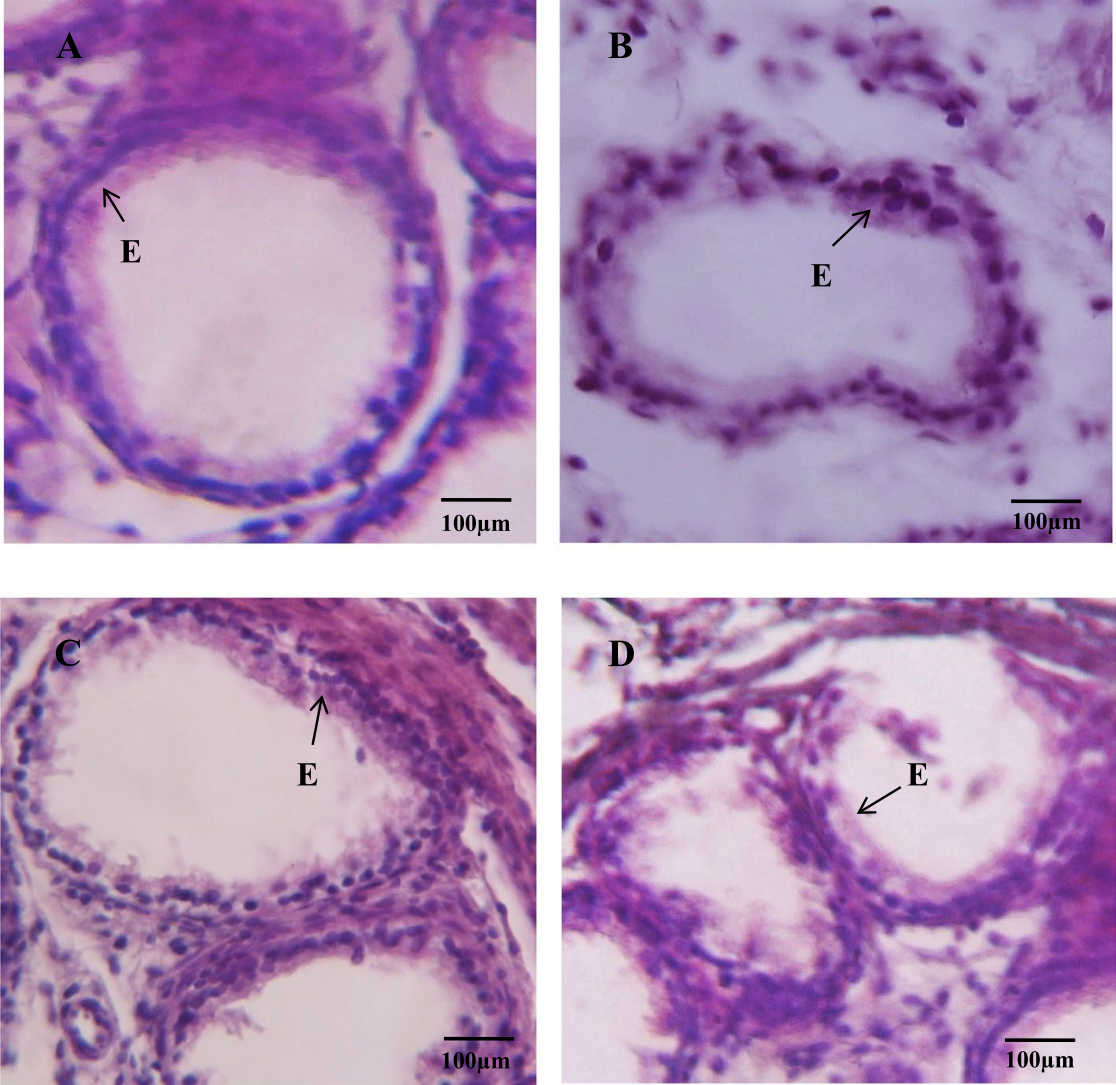
Photomicrograph of caput of the epididymis at PND 26 (H&E, 40X): (**A**) Control; showing normal morphology of caput epididymis, thin pseudostratified epithelium (B) IMD (1 mg/kg) group; showing reduced pseudostratified epithelium with empty lumen (**C**) IMD (5 mg/kg) group; showing empty lumen and reduced epithelium, (**D**) IMD (10 mg/kg) group; showing further distortion of the pseudostratified epithelium as compared to other two groups group. Stereocilia (St), Epithelium (**E**). Pictures were taken at Magnification 40X

Histomorphological investigation of caput epididymis in the control group exhibits a wider diameter, larger lumen and thin pseudostratified epithelium. The tubules of epididymis were compactly arranged and bounded by stroma as shown (Fig. 4A). Treatment with IMD (1 mg/kg) prenatally leads to degeneration of epididymal tubules. An increase in the interstitial space was seen along with a significant reduction in the stromal space surrounding the lumen (Fig. 4B). A significant decrease in lumen diameter was administered (*p* < 0.001) when all treated groups are compared with the control (Fig. 4C). The lumen diameter was decreased significantly in the IMD (10 mg/kg) group leading to a decrease in the thickness of the wall in comparison to the control group (Fig. 4D). A significant (*p* < 0.01) reduction in lumen diameter was noticed when G1 was compared with G3, while no change was observed between G1 and G2 (Table 3). There was also a significant difference (*p* < 0.001) in luminal diameter when G2 and G3 are compared. A significant (*p* < 0.001) lowering in epithelial height as compared to control was seen among all the IMD treatment groups. A significant reduction (*p* < 0.01, *p* < 0.001) was observed when G2 and G3 were compared to G1. Non-significant changes were noticed when G2 and G3 were compared.

### Cauda epididymis

**Fig. 5:**
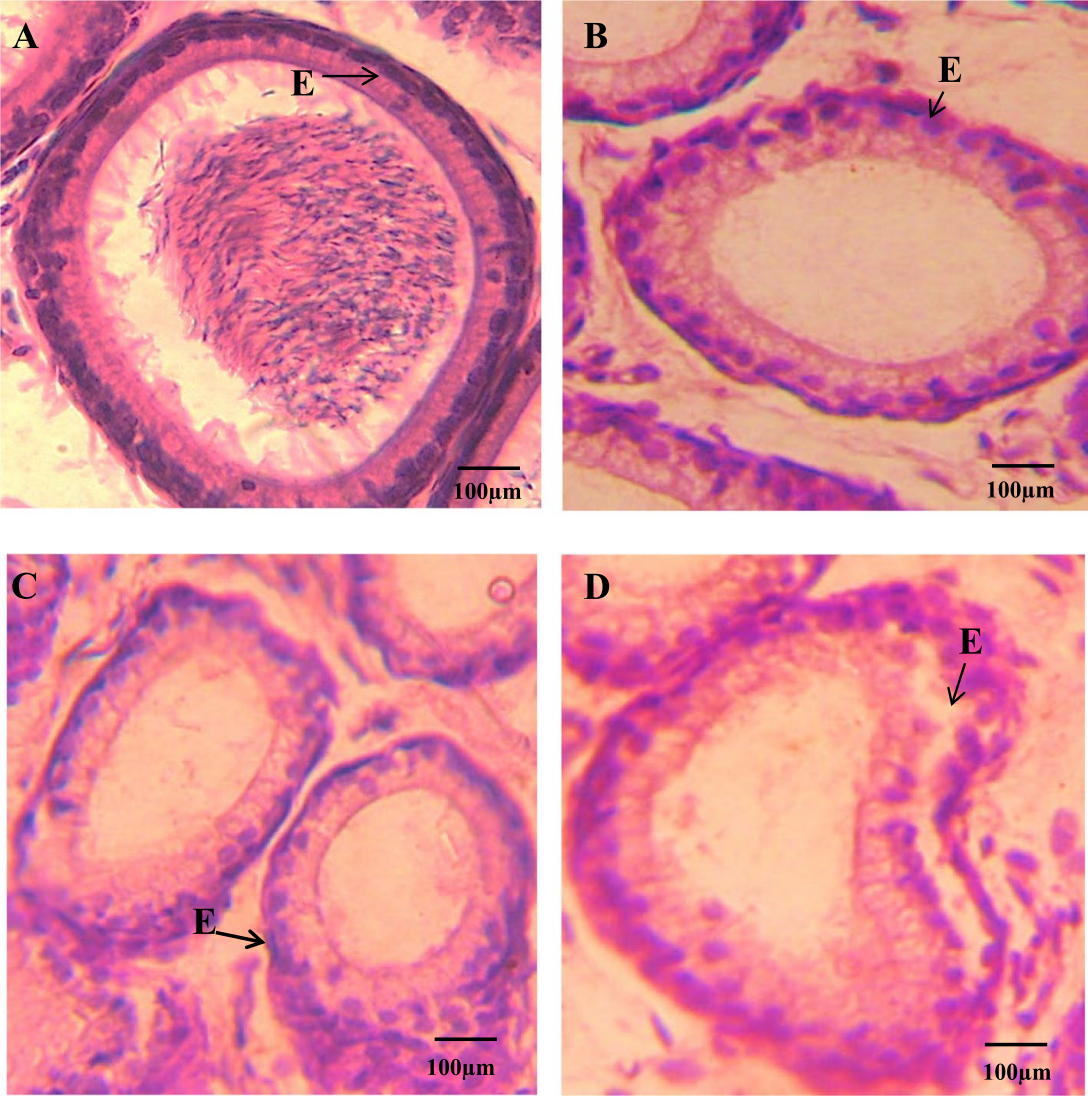
Photomicrograph (H&E, 40X) of the cross-section of cauda of epididymis: (**A**) Control; display typical morphology of cauda epididymis showing densely arranged tubules with thick epithelium (**B**) IMD (1 mg/kg) treated group; display slightly thick epithelium (**C**) IMD (5 mg/kg) of dose treated group; showing an irregular arrangement of tubules surrounded by stroma, the reduced epithelium (**D**) IMD (10 mg/kg) of a dose-treated group); showing a further decrease in the epithelium. Epithelium (**E**). Pictures were taken at Magnification 40X

The cauda segment of epididymis from the control group demonstrated normal morphology, with the presence of closely arranged tubules surrounded by stroma, and thick and pseudostratified epithelium, with a lumen filled with spermatozoa. Two types of cells were present, principal cells which extend from the basal lamina to the short lumen and the other one is basal cells located at the basal lamina as shown in Fig. 5. A significant decrease (*p* < 0.001) was noticed in the tubular diameter of cauda epididymis in the G3 group when compared with the control (Table 3). The non-significant difference was noticed when G1 and G2 were compared to the control group. In comparison to the control group, G2 and G3 groups showed a significant reduction (*p* < 0.05, *p* < 0.001) in the lumen diameter of the cauda epididymis. However, a highly significant difference (*p* < 0.001) was noticed, when G1 is compared to G3. Similarly, a comparison of G2 with G3 depicted a significant change (*p* < 0.01) in lumen diameter as shown in (Table 3). A significant decrease (*p* < 0.001) in epithelial height among all the IMD-treated groups when compared with the control. Non-significant differences were noticed when treatment groups were compared with each other.

## Discussion

The prevalent use of imidacloprid (IMD) leads to risk-vulnerable populations, including men and women of reproductive age [39]. Therefore, it is important to investigate the impact of IMD exposure on reproductive health and development. Many investigations have studied the impact of exposure on adult rats. However, our goal was to examine the impact of IMD exposure on male rats during the neonatal period to juvenile period on reproductive organs at levels pertinent for real-life scenarios set out in the current EU legislation and in general considered not detrimental to humans. Given the possible toxic effect of imidacloprid on male reproduction, we determined whether and how a repeated 26-day exposure from PND 1 to PND 26 affected antioxidant defence and persuaded impairment in testicular and epididymis tissue.

In the current investigation, a decrease in body and testicular weight was recorded in exposure groups. Many previous studies highlighted the decrease in body weight, testis and accessory organ weights of male rats exposed to different doses of IMD, which led to the decreased number of Leydig cells and is attributed to seminiferous tubule atrophy [40]. Previous studies also reported that prenatal exposure to IMD decreased the body weight and accessory organ weight in animals after the treatment with different doses [41]. The decrease in accessory organs' weight might be due to significantly lower testosterone (T) concentrations due to exposure to IMD, as accessory sex organs and epididymis need continuous stimulation of androgens for their normal growth and function [42].

The present study was also designed to check antioxidant enzyme status after IMD exposure in neonatal male rats. The ROS levels in IMD-treated groups increased significantly which is considered an indication of oxidative stress. Studies by [43] and [44] also indicated decreased CAT and SOD levels in the testis of IMD-treated rats. Other findings also showed increased antioxidant enzymes including CAT and SOD in the liver, kidney and brain of male rats after IMD administration [45]. In the present study, IMD treatment in neonatal male rats showed decreased levels of antioxidant enzymes, CAT and SOD in comparison to the control which is similar to previously published data [46].

In the present study, we evaluated the antioxidant enzyme profile to assess the effects of postnatal IMD exposure on male rats. Previous literature suggests that exposure to IMD causes a substantial increase in ROS production in testicular cells [47, 48]. Initiation of excessive ROS production also leads to alterations in antioxidant enzymes and subsequently to oxidative stress which is also considered responsible for IMD toxic mechanism [49]. The generation of ROS also leads to protein degradation, and LPO [50]. The increased free radicals as well as lower antioxidant enzymes may also cause cell disruption along with oxidative damage towards the plasma membrane [51]. ROS generally has deleterious effects on male reproductive organs which may at times result in reduced fertility, which can be demonstrated by increased disruptions in the germ cells of the testicular tissues [41].

Generally, the serum lipid profile increased during the postnatal period. We noticed that IMD (10 mg/kg dose) results in an increased level of cholesterol. Cholesterol is the foremost precursor of steroidogenesis and is made chiefly in the liver from HDL and LDL. Earlier data suggested that the administration of IMD and clothianidin increases the total cholesterol level in kidney tissues of adult and infant rats [52]. Similarly, Bal and coworkers have testified that clothianidin (pesticide) stimulates the biosynthesis of cholesterol in testis tissue [37]. The current study was parallel with previous findings and indicated the increased levels of cholesterol in testis tissues. IMD treatment may have a stimulating effect on the activity of the enzyme that is responsible for the transcription of cholesterol and therefore results in differences in levels of cholesterol.

The present study indicated an increase in triglyceride (TGs) levels at the 10 mg/kg dose of IMD. An earlier study similarly indicated the high TGs levels in liver rats by exposure to IMD, which results in a free radical generation that directly leads to ultra-structural changes in the liver [38]. There may be the generation of free radicals in the testis which are responsible for encouraging oxidative stress when exposed to IMD which is clear from corresponding ultra-structural and histological alterations in the observed rat testis.

Furthermore, we recorded significant decrease in HDL in all IMD-treated groups as compared to control and a slight increase in LDL in G2 and G3 compared to control. These results were in close agreement with a previous study in which high doses of IMD caused high LDL levels and low HDL levels in adult rat liver tissue [38].

The present study also depicted low testosterone concentrations in G2 and G3 (5 and 10 mg/kg) in developing male rats from PND 1 to PND 26. This decrease in the levels of testosterone is due to the inhibitory effect of IMD on testosterone production. Similar results (low testosterone concentrations) were also observed previously when different concentrations of IMD (8, 2 and 0.5 mg/kg) were exposed in developing male rats [24]. Previous data also suggested that the imbalance in the testosterone concentrations in both immature and mature rats after treatment with IMD chronically resulted in a lower number of Leydig cells [40, 53, 54]. Parallel results were also achieved for endosulfan, with different concentrations when male rats were chronically exposed [55].

In the present study, IMD exposure for 26 days resulted in reduced tubular diameter and epithelial height in all treated groups of IMD while an increase in lumen diameter and interstitial space which suggests an affected process of spermatogenesis. Previously, [40] it was reported that decrease in the weights of testis and accessory glands i.e., seminal vesicles and prostate, which also led to a low number of Leydig cells and atrophy in the seminiferous tubules after the treatment of IMD. Similar findings have also been observed after exposure to IMD leading to oxidative damage of the testis and apoptosis of spermatogonia [41, 56]. Low sperm count (oligozoospermia), reduced sperm morphology (teratozoospermia), and low sperm motility (asthenospermia) were evident in both immature and mature rats due to treatment with IMD [53, 57].

IMD caused a reduction in Leydig cell number in the testis along with cytoplasmic granulation and hypertrophy [56]. In the current study decreased testosterone concentrations were observed and this sexual imbalance is due to IMD's recognized impact on Leydig cells' degeneration or indirect nicotine-like oppressive effect on LH secretion through the pituitary gland [53, 57]. This might be because of the high polyunsaturated fatty acids and low antioxidant enzyme ability of male germ cells that make them vulnerable to oxidative stress and lipid peroxidation.

## Conclusion

Sub-chronic exposure to IMD during the neonatal to juvenile period may cause infertility in males via inducing testicular damage, decreasing testosterone production and increasing oxidative stress. It seems to disrupt the normal function of the testis in male rats and its detected biochemical effects, even if associated with increased imidacloprid dosage, specify the potential risks posed by this broadly used insecticide and point out the implication of protective actions and safety guidelines to reduce its usage. As residues of IMD can be found in regularly consumed food and water, and human exposure typically comprises a combination of numerous chemicals that can act in an additive or even synergistic mode, the influence of IMD and its mechanism of action of toxicity on reproductive organs warrants further investigation.

## Data Availability

The data generated in the current study is available from the corresponding author on reasonable request.
